# Novel antimicrobial/antioxidant *Eremurus luteus* root gum coating containing rosemary essential oil nanoemulsions for extension of chicken meat shelf life

**DOI:** 10.1002/fsn3.3295

**Published:** 2023-03-09

**Authors:** Dina Shahrampour, Seyed M. A. Razavi

**Affiliations:** ^1^ Department of Food Safety and Quality Control Research Institute of Food Science and Technology Mashhad Iran; ^2^ Center of Excellence in Native Natural Hydrocolloids of Iran Ferdowsi University of Mashhad Mashhad Iran

**Keywords:** chicken fillet, essential oil nanoemulsion, glucomannan, packaging

## Abstract

Herein, the effect of incorporation of rosemary essential oil (REO) nanoemulsions with the smallest (98.14 nm) and largest (148.04 nm) droplets' sizes at different concentrations (0%, 2%, and 4% v/v) in *Eremurus luteus* root gum (ELRG) coating solution on microbial, chemical, and sensory qualities of chicken fillets during cold storage was investigated. The results demonstrated a significant reduction in pH and TBA value and total viable microbial count (TVC) of chicken meat samples after using an active ELRG coating compared with the uncoated sample. Moreover, the properties of active ELRG coatings were more affected by the concentration of REO nanoemulsions than the size of their droplets. More antimicrobial and antioxidant activities were observed in coated samples containing 4% (v/v) REO nanoemulsions (L‐4 and S‐4). The highest and lowest pHs at the end of storage belonged to uncoated (6.89) and S‐4 coated (6.41) samples, respectively. Unlike the control sample (8th day), the microbial population in the active coated samples (>12th day) reached the threshold level (7 log CFU/g) later. The TBA value in the control and coated samples was 0.56 and 0.4–0.47 mg/kg after 12 days of cold storage, respectively. Increasing the REO nanoemulsion content from 2% to 4% (v/v) in the coating solution enhanced the score of sensory parameters such as odor, color, and total acceptance of the chicken meat, especially on the last day of cold storage. The obtained results suggested ELRG‐REO coatings as an effective strategy to delay the chemical and microbial deterioration of chicken meat fillets.

## Graphical abstract

Developed active *Eremurus luteus* root gum coatings containing REO nanoemulsions reduced lipid oxidation and microbial deterioration in chicken fillets and extended the shelf life at 4°C.

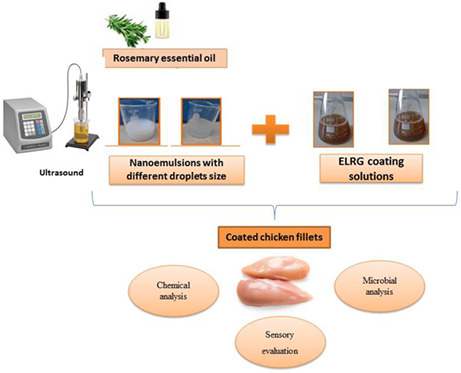

## 1 INTRODUCTION

Today, most people are reluctant to consume red meat due to the outbreak of cardiovascular disease all over the world. In contrast, poultry meat as a source of protein is one of the favorite foods in many countries, because of its low production cost, favorable taste, lower fat content, and high nutritional value (Latou et al., 2014). Poultry meat is highly susceptible to microbial and chemical spoilage due to high moisture and protein contents and high pH value and relatively has a short shelf life around 5 days in the refrigerator (Kerry et al., 2006).

Chicken meat is usually supplied in the market in the form of fillets, which will be more susceptible to microbial contamination due to the cutting of meat and removal of the skin during cold storage. Therefore, extending the shelf life of raw chicken meat is a severe challenge in the food industry, and applying novel preservation methods is necessary. In recent years, the active coating containing antimicrobial or antioxidant components for meat packaging has received much attention in various studies (Ala & Shahbazi, 2019; Latou et al., 2014; Noori et al., 2018). This approach could improve meat quality and safety by reducing microbial growth and delaying lipid oxidation. Due to the increasing consumer awareness about the environmental and health hazards of using plastic packaging for food, the tendency to use biodegradable packaging made from natural and non‐toxic polymers (proteins, polysaccharides, and lipids) increased (Lee et al., 2019). The coating with antimicrobial properties on the food surface through a slow release of active components during storage could limit microbial growth. Moreover, the incorporation of active components in coatings may be more beneficial than the direct use of them in food, as a reaction of antimicrobial material with food components increases or eliminates their bioactivity (Appendini & Hotchkiss, 2002). In addition, the release of active compounds from the structure of packaging on the food surface depends on different factors such as polymer type, active compounds concentration, and storage temperature (Janes & Dai, 2012). Among the compounds widely used in the preparation of active packaging, plant essential oils are the most popular.

Essential oils (EOs) are natural aromatic and volatile lipophilic plant extracts with antimicrobial and antioxidant properties and could be a suitable alternative to synthetic chemical preservatives (Prakash et al., 2015). According to previous studies, the direct incorporation of EOs in the polymeric matrix has some disadvantages, including volatilization of EOs during water evaporation because of the coarseness of its droplets and creating porous structures in coating or film (Norajit et al., 2010; Tastan et al., 2016). Accordingly, application of EOs in the form of “nanoemulsion” with tiny droplet sizes has different advantages including, high stability, enhancement of physicochemical properties, and improved biological properties through increasing the specific surface area and hence lowering the required amounts of the active component. Nanoemulsions are emulsions with particle sizes in the range of 2–200 nm that produced by high‐energy or low‐energy emulsification methods (McClements & Rao, 2011). Recently, some bio‐based coatings containing nanoemulsion of EOs have indicated good antimicrobial activity on different food surfaces such as chitosan/ carvacrol nanoemulsions (Severino et al., 2015), alginate/citrus EO nanoemulsions (Das et al., 2020), sodium caseinate/ginger EO nanoemulsions (Noori et al., 2018), pullulan/cinnamon EO nanoemulsions (Chu et al., 2020), and chitosan/*Zataria multiflora* Boiss or *Bunium persicum* Boiss EO nanoemulsions (Keykhosravy et al., 2022).

The genus of *Salvia rosmarinus* is an aromatic woody perennial plant that belongs to the mint family Lamiaceae and is known as rosemary. It is found abundantly in the Mediterranean and Asia regions but can cultivate in other world regions as an ornamental and aromatic plant (Yu et al., 2013). Rosemary is commonly used as a food flavoring agent or for different pharmacological purposes. The EO of rosemary is a colorless or pale yellow liquid with a characteristic plant odor and mainly consists of monoterpenes (Shahrampour & Razavi, 2022). Recently, antioxidant and antimicrobial properties of rosemary essential oil (REO) have been reported in some studies (Chraibi et al., 2020; Hassanzad Azar et al., 2019; Nieto et al., 2018; Ramadan et al., 2020).

The *Eremurus* (known as Serish or Cerish in Iran) belongs to the *Liliaceae* family and is geographically dispersed in various regions of Asian countries such as Iran. *Eremurus luteus* root gum (ELRG) is a good source of low‐cost and non‐toxic carbohydrates. One of the main heteropolysaccharides found in ELRG is glucomannan. According to our previous research, ELRG showed good film‐forming capacity and high solubility in water (Shahrampour & Razavi, 2023). As far as we know, the application of ELRG as a coating on food surfaces has received no attention. Moreover, the effect of particle size of active components in coating structure on food quality properties has not been investigated. Therefore, the present study aimed to evaluate the effect of ELRG coating containing different concentrations of REO nanoemulsions with various droplet sizes on microbiological, chemical, and sensory parameters of chicken breast fillet during cold storage.

## 2 MATERIALS AND METHODS

### 2.1 Materials

The various media such as PCA, MRS agar, and YGC agar were purchased from Liofilchem Company. REO was prepared from Exire Gole Sorkh Company. ELRG powder was donated by Salahi et al. in June 2021 and kept in a glass container in a dark and dry place. The identified compounds of ELRG are stated in Table 1. Glycerol, Tween 80, thiobarbituric acid, and other reagents were purchased from Merck Company. Fresh chicken breast fillets were provided from a local market in Mashhad and quickly transferred to the laboratory in a polystyrene box containing ice.

**TABLE 1:** *Eremurus luteus* root gum (ELRG) compounds according to the report of Salahi et al. (2021)

Compounds	Percentage (%)
**Total carbohydrate**	86.45
Glucose	50.42
Mannose	45.8
Fructose	2.07
Galactose	0.96
Galacturonic Acid	0.75
**Protein**	4.22
Uronic Acids	8.6
**Ash**	4.17
**Moisture**	6

### 2.2 Preparation of REO nanoemulsions

The nanoemulsions of REO essential oil were prepared according to the method of (Fattahi et al., 2020) with some modifications. The base emulsion was first formulated by aiding dropwise of REO and Tween 80 with a ratio of 1:0.5 (v/v) to 20 mL distilled water and homogenization by an Ultra‐Turrax at 10,000 *g* for 2 min. The nanoemulsions were obtained after sonication treatment of the emulsion at 20 kHz frequency, 200 W input power, and 30% amplitude for different times (2.5, 5 and 10 min) by an Ultrasonicator (FAPAN). The container of each nanoemulsion was kept in an ice bath to prevent increasing the temperature during sonication. All emulsions contained 10% (v/v) of REO.

### 2.3 Measurement of droplet size

The average droplet size (*z*‐average) of the REO nanoemulsions was determined by dynamic light scattering (DLS) technique (model NanoQ, Instrument VASCO). Before the analysis, nanoemulsions were diluted with deionized water to 1:100 to avoid multiple scattering effects (Noori et al., 2018). This test was done in triplicate at 25°C.

### 2.4 Preparation of coating solution

1.5 g of the ELRG powder was mixed with 100 mL distilled water and continuously stirred at 85°C and 800 rpm for 30 min. Also, glycerol was added at 30% (w/w) of ELRG powder as a plasticizer agent. Afterward, the smallest and largest droplet sizes of REO nanoemulsions at two concentrations (2% and 4%, v/v) were added with constant agitation at room temperature for 20 min. The final solutions were applied for coating chicken breast fillets in the next section.

### 2.5 Coating of chicken breast fillet

The fresh chicken breast fillets were aseptically cut in the same size weighing about 20 g. The coated layer was formed on the surface of each chicken fillet's pieces after dipping into the ELRG coating solutions for 2 min and drying at room temperature for 10 min. Also, a control sample was prepared after dipping in distilled water at similar conditions. All samples were placed in polystyrene (PS) containers and kept in a refrigerator for 12 days and the following analyses were carried out on the 1st, 4th, 8th, and 12th days of storage.

### 2.6 Chemical analyses

#### 2.6.1 pH measurement

The pH was measured using AOAC (2005) method. Ten grams of each chicken fillet sample was added to 90 mL of distilled water and homogenized with a mixer for 2 min. The pH of each sample was measured by digital pH meter electrodes at room temperature.

#### 2.6.2 Thiobarbituric acid determination

Thiobarbituric acid (TBA) was estimated by using the colorimetric method as described by Majdinasab et al. (2020). Briefly, 200 mg of the homogenized sample of chicken fillet was transferred to a container containing 25 mL of 1‐butanol and mixed well. TBA reagent was prepared by dissolving 200 mg of TBA in 100 mL of 1‐butanol solvent and after filtration kept in a refrigerator. Then, 5 mL of previous sample mixture was added to a clean and dry test tube containing 5 mL of TBA reagent. All tubes were placed in a hot water bath at 95°C for 1 h. After cooling the tubes at ambient temperature, the absorbance at 530 nm was determined by a spectrophotometer. The control tube only contained TBA reagent. According to the following formula, the amount of TBA in terms of mg of malondialdehyde per kg of chicken fillet was calculated.

$$\mathrm{TBA}=50\times \left[\left(A_{s}–A_{c}\right)/200\right]$$

*A*_c_, absorbance of the control; *A*_s_, absorbance of the sample.

### 2.7 Microbiological analyses

The counts of total mesophilic, psychrophilic, molds and yeasts, and lactic acid bacteria on chicken fillets were evaluated during cold storage. Briefly, 10 g of chicken fillets were transferred aseptically into individual stomacher bags containing 90 mL of sterile NaCl solution (8.5 g/L) and homogenized in a stomacher for 1 min. Serial dilutions were made, and pour plate culture was prepared on PCA and MRS medium. The PCA plates for determining total viable mesophilic and psychrophilic microorganisms count were incubated at 37°C for 24 h and 10°C for 10 days, respectively. Also, the MRS plates for enumeration of lactic acid bacteria were incubated at 37°C for 2 days. The YGC plates were used to evaluate the count of molds and yeasts in samples and incubated at 25°C for 72 h. After the incubation period, the microbial load of chicken fillet samples was reported as log CFU/g.

### 2.8 Sensory evaluation

The sensory attributes of chicken fillet samples were analyzed by seven members trained panel. The sensory properties of different treatments such as color, odor, and general acceptance were scored using a 5‐point descriptive hedonic scale (1 = dislike extremely and 5 = like extremely).

### 2.9 Statistical analysis

All results were presented as the mean ± standard deviation. The statistical analysis of data was performed by analysis of variance (ANOVA), and the comparison of means was done by Duncan test at a significance level of 5% using SPSS software (version 21).

## 3 RESULTS AND DISCUSSION

### 3.1 Droplet size of REO nanoemulsions

**FIGURE 1:**
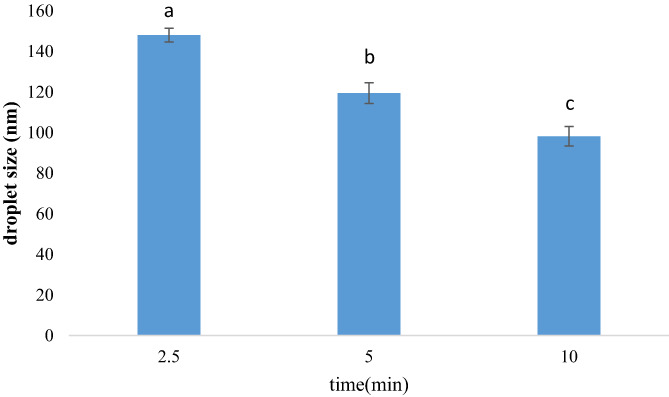
Effect of different times of ultrasound treatment on droplet size of rosemary essential oil (REO) nanoemulsions.

The droplet size of REO emulsions measured by DLS is depicted in Figure 1. The results indicated that increasing the time of sonication treatment from 2.5 to 10 min significantly reduced the particles size of REO emulsion (10% v/v) from 148.04 to 98.14 nm (*p* < .05). These nanoemulsions with different droplet sizes were used as active components in the next stage for the fabrication of active ELRG coatings. The ELRG coatings containing REO nanoemulsions with the smallest and largest size were named S and L in this study, respectively. In a similar study, Ghosh et al. (2013) formulated stable basil oil nanoemulsion (6% v/v) with droplet diameter of 29.3 nm by ultrasonic emulsification for 15 min. In another study, the droplet size of ginger EO nanoemulsion with a concentration of 5% (v/v) after the sonication process for 5 min was measured as 57 nm (Noori et al., 2018). Chu et al. (2020) observed a decline in droplet size of cinnamon EO emulsions (6% v/v) from 231 to 60 nm after an increase in sonication time from 2.5 to 10 min. Hasheminya and Dehghannya (2021) reported a larger droplet size for *Salvia mirzayanii* EO nanoemulsion (168 nm) after 10 min sonication compared with our nanoemulsion in this research. It could be concluded that the EO type and their concentration in emulsions and also time and power of sonication treatment are effective factors in the droplet size of nanoemulsions.

### 3.2 Chemical properties of chicken fillet samples

#### 3.2.1 pH

**FIGURE 2:**
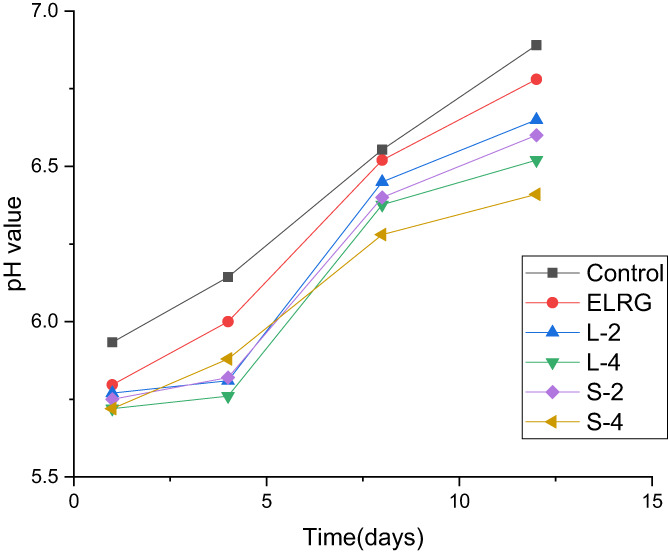
Effect of the ELRG coating containing rosemary essential oil (REO) nanoemulsion on pH changes of chicken fillets during cold storage. (Control: sample without coating, ELRG: sample coated with *Eremurus luteus* root gum, L‐2: sample coated with ELRG containing the largest droplet size of nanoemulsion with 2% v/v concentration, L‐4: sample coated with ELRG containing the largest droplet size of nanoemulsion with 4% v/v concentration, S‐2: sample coated with ELRG containing the smallest droplet size of nanoemulsion with 2% v/v concentration, S‐4: sample coated with ELRG containing the smallest droplet size of nanoemulsion with 4% v/v concentration).

The effect of coating on pH changes of chicken fillet samples during cold storage was exhibited in Figure 2. The results revealed no significant difference between the samples on the first day of storage and the pH varied from about 5.72 to 5.93 (*p* < .05). Moreover, the pH value of all treated chicken fillets increased during the storage time. The enhancement of the pH value of chicken fillet samples can be attributed to the production of basic compounds such as ammonia, primary amines, and secondary amines by food spoilage aerobic microorganisms on meat surfaces (Cai et al., 2014). The coated samples with higher REO nanoemulsion concentrations including S‐4 and L‐4 had lower pH than other samples at the end of storage time. The highest and lowest pH on the 12th day belonged to the uncoated control sample (6.89) and the coated sample with S‐4 coating containing 4% REO nanoemulsion (6.41), respectively. The lower pH value in active coated samples might be due to the antimicrobial and antioxidant activity of REO components that delayed microbial growth and protein oxidation. These results were consistent with research about the coating of chicken fillets with sodium caseinate nanocomposite containing cinnamon essential oil (Ranjbaryan et al., 2018). Majdinasab et al. (2020) investigated the effect of adding two types of essential oils thyme and savory in basil coating solution on the shelf life extension of chicken fillets. Their results showed that the sample coated with basil solution containing 2% of thyme had the lowest pH at the end of cold storage time. Applying konjac glucomannan/carrageenan coating incorporated camellia oil on chicken meat delayed the upward trend of pH value (Zhou et al., 2021).

#### 3.2.2 Lipids oxidation

**FIGURE 3:**
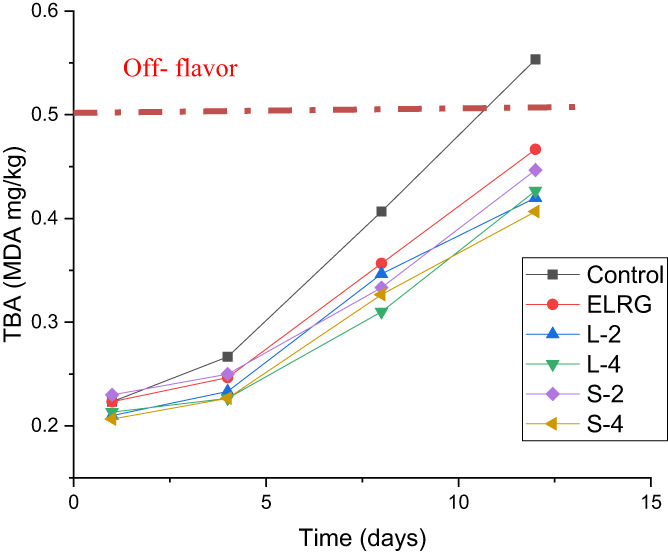
Effect of ELRG coating containing REO nanoemulsions on TBA index of chicken fillets during cold storage. (Control: sample without coating, ELRG: sample coated with *Eremurus luteus* root gum, L‐2: sample coated with ELRG containing the largest droplet size of nanoemulsion with 2% v/v concentration, L‐4: sample coated with ELRG containing the largest droplet size of nanoemulsion with 4% v/v concentration, S‐2: sample coated with ELRG containing the smallest droplet size of nanoemulsion with 2% v/v concentration, S‐4: sample coated with ELRG containing the smallest droplet size of nanoemulsion with 4% v/v concentration).

Lipid oxidation is one of the leading causes of bad taste in meat products, especially when the meat has high unsaturated fatty acids and is stored under aerobic conditions. Lipids oxidation is commonly assessed by the TBA index value, which indicates the amount of the by‐product of oxidation such as aldehydes and expresses in mg of malondialdehyde (MDA) per kg of meat. In this regard, the oxidation of lipids in chicken meat leads to the production of compounds such as aldehydes, ketones, alcohol, and acids, changes the flavor of the meat, and reduces its nutritional value (Majdinasab et al., 2020). The threshold of lipid oxidation and off‐flavor development in the food according to the TBA index is reported as more than 0.5 mg MDA/kg. The maximum tolerable limit of TBA without any adverse effect on chicken quality is estimated 4 mg MDA/kg (Majdinasab et al., 2020). As depicted in Figure 3, a similar increase in the amount of TBA index was observed in all chicken fillet samples during the storage time. There was no significant difference between the TBA index of the uncoated control sample and coated samples on the 4th day (*p* > .05). The highest significant difference between the control and other samples was observed at the end of cold storage (*p* < .05). The TBA values varied from 0.40 to 0.47 MDA mg/kg for coated samples at the end of storage time. The incorporation of REO nanoemulsions in the coating solution could retard the production of secondary products of lipid oxidation in chicken meat. In addition, the coating of samples with REO nanoemulsions with two droplet sizes and concentrations was not significantly different in this regard (*p* > .05). The TBA value in the control sample exceeded 0.5 mg/kg on Day 10 while in coated samples was lower than the threshold value. These results revealed the antioxidant effect of the REO nanoemulsions in coatings due to the presence of monoterpene compounds in preventing the rapid increase of MDA content in chicken fillets (Shahrampour & Razavi, 2022). In addition, the polysaccharide‐based coating with its inhibitory properties against oxygen gas can play a role in delaying the oxidation of lipids. In another study, an increase in MDA in chicken fillet samples coated with sodium caseinate was reported and the lowest level of TBA on the 12th day of cold storage was 0.12 mg/kg related to a coating containing 2.5% cinnamon EO (Ranjbaryan et al., 2018). In the study of Noori et al. (2018), the amount of TBA value in all coating chicken samples to be 0.02 mg/kg which reached 0.06 mg/kg after 12 days. Also, there was no significant difference between the TBA index of chicken fillet samples coated with sodium caseinate‐containing emulsion and nanoemulsion of ginger EO. They also stated that the antioxidant properties of this type of coating were weaker than its antimicrobial properties. Jouki et al. (2014) determined the amount of TBA index at the end of the 18th day of cold storage in control and coated fish fillets with quince seed mucilage coating containing oregano and thyme EOs as 0.95, 0.43, and 0.49 mg/kg, respectively. In Zhou et al. (2021) research, the TBA values in chicken samples after covering with konjac glucomannan/carrageenan coating containing camellia oil ranged from 0.69 to 1.4 after 10 days of cold storage. Keykhosravy et al. (2022) observed different reduction of TBA index after adding 1% (v/v) of *Zataria multiflora* Boiss (17.3%) and *Bunium persicum* Boiss (7.03%) EOs nanoemulsions in chitosan coatings of turkey breast fillets compared to control sample at the end of 15 days storage at refrigerator.

In general, the antioxidant activity of essential oils is related to various mechanisms such as preventing the formation of free radicals, limiting the transfer of metal ion catalysts, decomposing peroxides, and interacting with free radicals. Cold storage was also reported as a factor in delaying the fat oxidation process in all samples. Fernandez et al. (1997) stated that the TBA index could probably report at incorrectly rate due to several interactions of MDA with amino acids, proteins and glucose, and other compounds in meat products.

### 3.3 Microbial quality of chicken fillet samples

**FIGURE 4:**
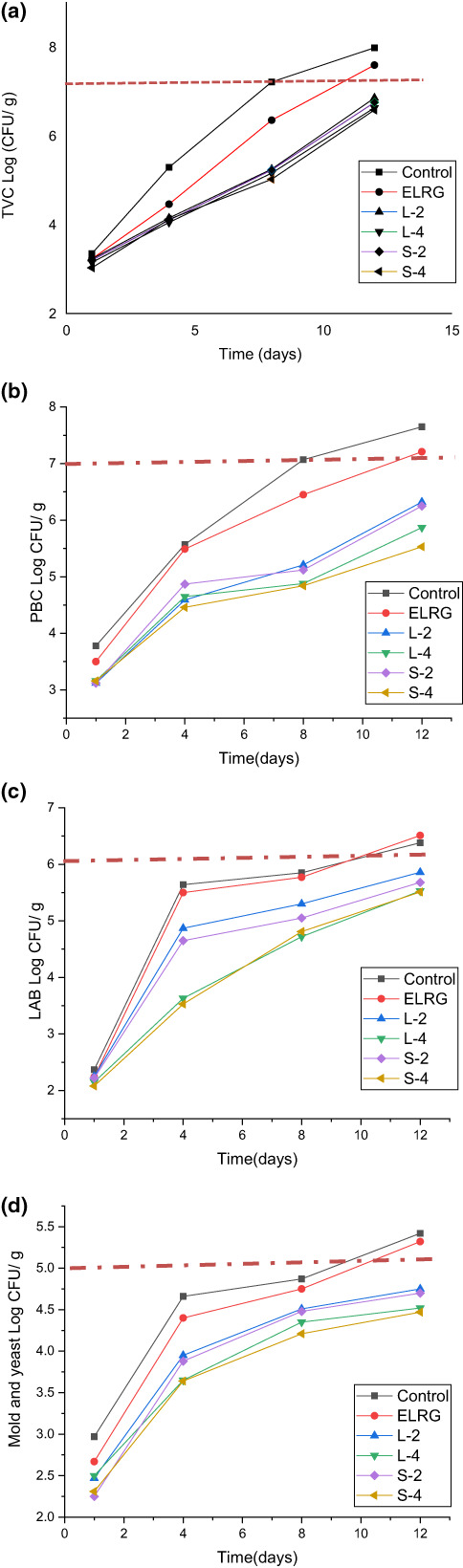
Effect of ELRG coating containing REO nanoemulsions on microbial count (CFU/g) in chicken fillets during cold storage: (a) Total viable mesophilic count (TVC), (b) psychrophilic, (c) lactic acid bacteria, and (d) molds and yeasts. (Control: sample without coating, ELRG: sample coated with *Eremurus luteus* root gum, L‐2: sample coated with ELRG containing the largest droplet size of nanoemulsion with 2% v/v concentration, L‐4: sample coated with ELRG containing the largest droplet size of nanoemulsion with 4% v/v concentration, S‐2: sample coated with ELRG containing the smallest droplet size of nanoemulsion with 2% v/v concentration, S‐4: sample coated with ELRG containing the smallest droplet size of nanoemulsion with 4% v/v concentration).

The effect of active ELRG coating on the microbial quality of chicken fillets during the storage time is presented in Figure 4. The count of 7 log CFU/g was reported as a critical microbial load for the start of microbial food spoilage (Senter et al., 2000). The results of total viable count (TVC) showed a significant increase in all sample treatments by increasing storage time at 4°C. Moreover, microbial load in uncoated and ELRG coated samples reached 7 log CFU/g sooner than in other samples (Figure 4a). TVC value of the control sample exceeded to threshold level on the 8th day of cold storage, while in active coated samples exceeded to threshold level after the 12th day. Incorporating different levels of REO nanoemulsions in ELRG coating solution led to microbial inhibition activity on the chicken meat surfaces. Generally, no significant difference was demonstrated among TVC values of active coated chicken samples. Compared with uncoated treatment, applying ELRG, L‐2, L‐4, S‐2 and S‐4 coatings lowered the TVC in chicken fillets by 4.88%, 14.16%, 16.92%, 15.41%, and 17.54%, respectively. Microbial growth inhibition was possible due to bioactive substances such as polyphenols in REO and a better influx of its nanoemulsion form with smaller size in microbial cell membrane. Besides, gas barrier property of polysaccharide‐based ELRG coating could limit oxygen accessibility for aerobic microorganisms. In Zhou et al. (2021) study the TVC of chicken fillet after covering with konjac glucomannan/carrageenan containing 3.5% camellia oil exceeded to threshold level on 10th day of chilled storage and TVC reduction was 19.73% in comparison with the control sample.

Psychrophilic bacteria such as *Pseudomonas*, *Aeromonas*, *Shewanella*, and *Flavobacterium* are the leading cause of food spoilage under cold storage conditions. The results of this study revealed a significant increase in psychrophilic microorganism's count on the surface of chicken fillets especially for the control sample during the storage at 4°C (Figure 4b). The total psychrophilic bacterial count (PBC) in the control sample reached 7 CFU/gr after 8 days of cold storage while their counts in all active ELRG‐coated samples were lower than threshold even after 12 days. Moreover, the results demonstrated that by increasing the concentration of REO nanoemulsions in the ELRG‐coating solution, antimicrobial activity increased. The highest and lowest PBC levels at the end of storage time belonged to the control and coated samples containing 4% of REO nanoemulsions, respectively. The active coatings such as L‐2, L‐4, S‐2, and S‐4 could reduce PBC in comparison with uncoated sample by 17.38%, 23.26%, 18.3%, and 27.71%, respectively. In another study, the effect of antimicrobial compounds such as lysozyme mixed with rosemary and oregano EOs on vacuum‐cooked chicken shelf life was investigated. Their results exhibited the ability of active components to extend the shelf life of cooked chicken fillets by more than 7 days compared to control (Ntzimani et al., 2010). Similarly, Fernández‐Pan et al. (2014) reported the successful effect of oregano EO in whey protein coating compared to clove EO in reducing the microbial count of chicken fillets during 13 days of cold storage. The observed antimicrobial effects of active coatings could be related to many factors such as the type and concentration of EOs, the sensitivity of the microbial groups, the type of food sample, and storage condition in an experiment. Generally, the antimicrobial efficacy of EOs depend on their phenolic components' activity on protein binding and enzymes inhibition (Chraibi et al., 2020; Dávila‐Rodríguez et al., 2019; Jiang et al., 2011).

Also, a significant increase was observed in the count of lactic acid bacteria (LAB) in all stored chicken fillets after 4 days (Figure 4c). Anyway, the count of these bacteria was less than 7 log CFU/g on 12th day in control and coating samples. By increasing REO nanoemulsions content in ELRG coating to 4%, the LAB count of chicken meat reduced by 13.32%–13.63% compared with control sample. Contrary to these results, Fernández‐Pan et al. (2014) reported no effect of whey protein isolate coating containing 2% oregano or clove essential oil on reducing a load of LAB of chicken fillets to less than 7 CFU/g at the end of the 13th day of storage at 4°C.

In addition, the population of molds and yeasts demonstrated similar trends during cold storage. Unlike the control and ELRG sample, the count of molds and yeasts was less than 5 logs CFU/ml in the coated samples containing REO nanoemulsions on the 12th day of storage. These results revealed the positive efficacy of active ELRG coating containing REO nanoemulsions on the decline of microbial growth in the chicken fillet samples. On the other hand, no significant difference was observed among the population of molds and yeasts of chill‐stored coated chicken fillets including various droplet size of REO nanoemulsions. Noori et al. (2018) similarly reported that the use of 6% ginger EO nanoemulsion in the solution of sodium caseinate led to a significant reduction in the count of molds and yeasts in coated chicken fillets compared with the uncoated sample. Keykhosravy et al. (2020) considered the type of nanoemulsion to be effective in the occurrence of antimicrobial activity. They observed more antimicrobial activity of coatings loaded with 1% *Zataria Multiflora* Boiss EO nanoemulsions than *Bunium persicum* Boiss EO nanoemulsions on turkey meat surfaces. The highest reduction rate of total viable bacteria (2.06 log CFU/g), total psychrophilic (2.59 log CFU/g), lactic acid bacteria (2.51 log CFU/g), and yeast, and mold count (2.10 log CFU/g) were obtained in chitosan‐loaded nanoemulsion of *Zataria Multiflora* Boiss EO 1% in comparison with uncoated samples.

### 3.4 Sensory attributes of chicken fillet samples

**FIGURE 5:**
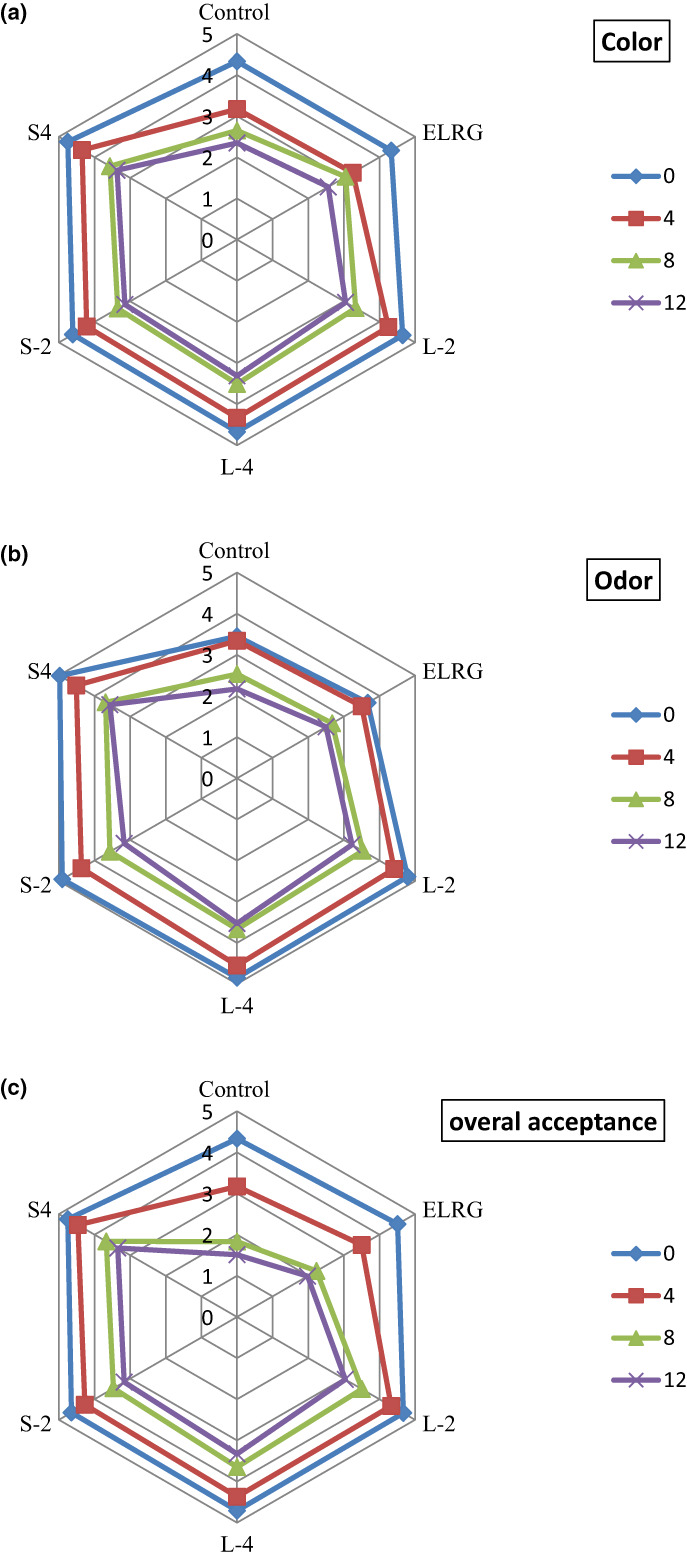
Effect of ELRG coating containing REO nanoemulsions on sensory attributes such as color, aroma, and overall acceptance of chicken fillets during 12 days of cold storage (Control: sample without coating, ELRG: sample coated with *Eremurus luteus* root gum, L‐2: sample coated with ELRG containing the largest droplet size of nanoemulsion with 2% v/v concentration, L‐4: sample coated with ELRG containing the largest droplet size of nanoemulsion with 4% v/v concentration, S‐2: sample coated with ELRG containing the smallest droplet size of nanoemulsion with 2% v/v concentration, S‐4: sample coated with ELRG containing the smallest droplet size of nanoemulsion with 4% v/v concentration).

The sensory properties of foods are an important factor in consumer acceptance. Microbial spoilage and lipid oxidation cause adverse changes in the smell, color, and taste of meats, which reduce their shelf life (Brannan, 2009). The sensory properties of stored chicken meat samples such as color, odor, and overall acceptance scored by trained panelists. This analysis showed a reduction in sensory scores with increasing cold storage time (Figure 5). On the first day of evaluation, there was no significant difference in the color properties of various active coated samples (Figure 5a). The color score of control sample was lower than three at the end of the storage period while coated samples were containing 2% and 4% of REO nanoemulsions still scored higher than three. On the other hand, the lowest odor score was assigned to both control and coated sample with ELRG during the cold storage. The addition of REO nanoemulsions as volatile compounds to ELRG coating solution could improve odor of chicken fillets during the storage period. The 4% REO nanoemulsions (S‐4 and L‐4) based coated samples showed the highest odor score on the 12th day. The increasing of REO nanoemulsions content in the coating solution enhanced the total acceptance score of the chicken fillet samples especially on the last day of chilled storage. Generally, the total acceptance score for tested samples especially for control sample decreased with increasing storage time due to the development of microbial and chemical spoilage. These results were consistent with the results of the microbial evaluation section, which determined the microbial shelf life of more than 12 days in the sample containing 2% and 4% REO nanoemulsions and eight days in the control sample. Moreover, the results of the sensory evaluation confirmed the results of the chemical analysis of coated and uncoated chicken fillets after 12 days of chilled storage. According to the chemical analysis, the shortest and longest shelf life belonged to control sample (10 days) and coated samples (>12 days), respectively. Generally, the presence of REO in the coating solutions produced an acceptable odor and appearance on the surface of chicken fillet samples for panelists. The applying EOs in the form of nanoemulsion in coating could reduce its severe odor and bad effect on panelists. In this regard, Noori et al. (2018) reported that chicken fillet samples with sodium caseinate coating containing 3% and 6% nanoemulsions of ginger EO showed the highest total acceptance score at the end of the 12th day of the storage period. Also, uncoated and coated samples without EO were removed from the evaluation in the following days on the 8th day due to their sticky surface and unpleasant odor. Ranjbaryan et al. (2018) considered adding 2.5% and 5% cinnamon EO to the sodium caseinate coating solution as effective in improving the sensory properties of chicken fillets. Moreover, the type of EO affected on the score of sensory parameters and the overall acceptance of panelists. For example, basil seed gum coating containing 1% and 2% of thyme EO provided higher sensory scores in color, odor, and total acceptance than savory EO for chicken fillets. Also, increasing in concentration of both EOs extended the shelf life of chicken meat up to 16 days (Majdinasab et al., 2020). According to Zhou et al. (2021) research, the sensory attributes of chicken fillet improved after the use of 3.5% of camellia EO in konjac glucomannan/carrageenan coating and its shelf life was prolonged up to at least 10 days.

### 3.5 Correlation between chemical and microbial parameters

As indicated in Table 2, a strong correlation was found between microbiological (TVC, PBC, LAB, Mold/yeast) and chemical (pH and TBA) parameters of all tested samples. For example, lipid oxidation and total microbial load of chicken fillets had a good correlation (0.961). In addition, a high correlation between pH value and TVC or TBA was observed. This observation supported further the role of specific spoilage microorganisms in the deterioration of chicken meat. Aerobic microorganism's activity on meat surfaces leads to the production of basic amins and results in an increase in pH. Although microorganisms are the main reason for meat spoilage, other mechanisms such as lipid oxidation could accelerate its deterioration. There was a high correlation between microbial parameters such as TVC, PBC, LAB and mold/yeast of all coated and uncoated samples. In similar, Assanti et al. (2021) observed positively correlated among microbiological, chemical, and sensory parameters of chicken burgers after coating with chitosan and vacuum packaging during storage at refrigerator.

**TABLE 2:** Pearson's correlation coefficients (*r*) among chemical and microbiological parameters of chicken fillet samples coated by ELRG/REO nanoemulsions.

Variables	pH	TBA	TVC	PBC	LAB	Mold/yeast
pH		0.959	0.958	0.894	0.846	0.875
TBA	0.959		0.961	0.882	0.803	0.818
TVC	0.958	0.961		0.958	0.902	0.912
PBC	0.894	0.882	0.958		0.941	0.955
LAB	0.846	0.803	0.902	0.941		0.983
Mold/ Yeast	0.875	0.818	0.912	0.955	0.983	

Abbreviations: LAB, lactic acid bacteria; PBC, psychrophilic bacteria counts; TVC, total viable counts.

## 4 CONCLUSIONS

The preparation and using of active *Eremurus luteus* root gum (ELRG) coatings containing REO nanoemulsions with different droplet sizes and concentrations on chicken fillets were investigated. Increasing ultrasonication time was an effective method for decreasing the droplet size of REO nanoemulsion. The present study revealed antimicrobial and antioxidant activity of ELRG coating incorporated with REO nanoemulsions which could extend the shelf life of chicken fillets at 4°C. The antimicrobial and antioxidant efficiency of REO nanoemulsions in the coating structure was affected by concentration to size. The lowest pH, TBA value, and total microbial count were obtained in coated chicken meat sample covered with ELRG coating containing the highest REO nanoemulsions content (4% v/v) at the end of storage time. Moreover, the active coated chicken fillets achieved a higher score for their sensory parameters than the uncoated sample. A strong correlation between chemical and microbial parameters of chicken samples during storage time was confirmed. This research revealed the potential of REO nanoemulsions especially with the smallest droplet size and highest concentration in the ELRG coating solution to prolonging chicken fillets shelf life during cold storage.

## CONFLICT OF INTEREST STATEMENT

The authors declare that there is no conflict of interest regarding the publication of this paper.

## ETHICAL APPROVAL

Ethics approval was not required for this research.

## Data Availability

The data that support the findings of this study are available from the corresponding author upon reasonable request.
